# Improvement in Glycemic Metrics With the First and Second Continuous Glucose Monitoring (CGM) Sensor Use in Indian Patients With Type 2 Diabetes Mellitus

**DOI:** 10.7759/cureus.82061

**Published:** 2025-04-11

**Authors:** Vipul Gupta

**Affiliations:** 1 Diabetes and Endocrinology, Gupta Ultrasound and Heart Care Centre, New Delhi, IND

**Keywords:** abbott freestyle libre, continuous glucose monitoring, diabetes control, standard blood glucose monitoring, time-in-range, type 2 diabetes mellitus

## Abstract

Background: Continuous glucose monitoring (CGM) provides the real-time monitoring of glycemic fluctuations. Better control of hemoglobin A1C (HbA1c) levels in patients with type 2 diabetes mellitus (T2DM) using Abbott FreeStyle Libre (Chicago, Illinois, United States) was reported. This study evaluated the glycemic outcomes in T2DM patients using Abbott FreeStyle Libre in Indian settings.

Methods: In this single-center retrospective study, data was collected from T2DM patients aged ≥18 years prescribed with Abbott FreeStyle Libre at Gupta Ultrasound and Heart Care Centre, New Delhi, India. The first application was considered the first use of FreeStyle Libre. The measurements were obtained from a sensor that operated continuously for as long as 14 days. The second application meant patients were using FreeStyle Libre for the second time continuously for 14 days following a break of two weeks after the first application. Time-in-range (TIR) is the percentage of time that a person spends with their blood glucose levels in a recommended target range. Time-below-range (TBR) is the time spent with blood sugar lower than the recommended range. Time-above-range (TAR) is the time spent above the recommended range. Relationship between TIR and various demographics/CGM metrics were analyzed.

Results: Overall, 649 and 60 patients were included from the first application and the second application, respectively. The average duration of DM was 10-15 years in most patients, with hypertension being the predominant comorbidity. TIR negatively correlated with lower HbA1c (r=−0.547; p<0.001), lower average glucose (r=−0.790; p<0.001), and TAR (r=−0.951; p<0.001) and positively correlated with TBR (r=0.190; p<0.001). TAR, TBR, and HbA1c were identified as significant predictors of TIR.

Conclusion: TIR from FreeStyle Libre showed a meaningful association with glycemic control, which could aid in optimizing treatment plans and improving clinical outcomes of T2DM patients.

## Introduction

Diabetes mellitus (DM) is projected to affect approximately 530 million adults globally, with a prevalence of 10.5% among adults aged 20-79 years. Type 2 DM (T2DM) constitutes roughly 98% of DM diagnoses worldwide, with notable variations across different countries [1]. Research analyzing the global epidemiology of T2DM predicted that the global prevalence of T2DM is expected to increase to 7,079 individuals per 100,000 by 2030, indicating a persistent upward trend across all regions worldwide, where lower-income countries have concerning trends of its increasing prevalence [2]. A study on the epidemiology of DM (Indian Council of Medical Research-India Diabetes; ICMR-INDIAB-17) between 2008 and 2020 in India observed that 101 million individuals had DM, which is expected to exceed 134 million by 2045, with approximately 57% of individuals being undiagnosed. The risk factors include ethnicity, age, obesity, physical inactivity, unhealthy diet, and behavioral habits, along with genetic and family history [3]. As per the ICMR-INDIAB study, the overall weighted prevalence of DM and pre-DM in individuals from rural and urban areas were 11.4% and 15.3%, respectively, suggesting the increasing epidemic of DM in India and warranting the need to arrest the upward trend [4].

Continuous glucose monitoring (CGM) devices offer significant value in both clinical and research contexts due to their ability to provide continuous data. Originally designed to enhance clinical care, CGM devices surpass conventional testing by delivering more frequent glucose measurements through a sensor inserted into the subcutaneous interstitial space [5]. The CGM system, FreeStyle Libre (Abbott, Chicago, Illinois, United States), was introduced in the United Kingdom in 2015. It marks the first generation of its kind, offering users an interstitial glucose value by scanning a glucose sensor using a reader device or compatible mobile phone. The FreeStyle Libre Pro system was further introduced in November 2020. This trial showed that replacing conventional self-monitoring of blood glucose with the innovative flash sensor-based CGM method can significantly reduce the time spent in hypoglycemia without any concurrent deterioration in hemoglobin A1C (HbA1c) levels [6]. Furthermore, a recent prospective study demonstrated that the second-generation factory-calibrated FreeStyle Libre System improved the analytical accuracy performance compared with plasma venous blood glucose reference in adult and pediatric patients. The system performed well in the hypoglycemic range with 94.3% of the results in the adult population and 96.1% of the data in the pediatric population being within the reference range of 15 mg/dL [7].

Several recent observational studies reported the use of FreeStyle Libre resulting in a decrease in HbA1c levels, a reduction in severe hypoglycemic events, and a decrease in the incidence of diabetic ketoacidosis [8,9]. In a meta-analysis conducted in 2022, 25 studies reported a significant and sustained reduction in HbA1c level in adult and pediatric patients with type 1 DM (T1DM) and in adult patients with T2DM who used the FreeStyle Libre system as part of their DM care [10]. A recent study conducted in 2022 showed that the use of the FreeStyle Libre Pro in patients with T1DM resulted in significantly lower HbA1c levels than those who monitored their blood glucose levels with the use of fingerstick testing [11].

This study aims to investigate the changes in glucose monitoring metrics after the initial and subsequent use of the FreeStyle Libre in patients with T2DM from India. The Indian demographic presents a unique set of challenges in DM care, including variations in lifestyle, dietary habits, and genetic predispositions. This study attempts to explore and quantify the effect of FreeStyle Libre on glucose monitoring metrics, providing evidence-based recommendations for its use in T2DM management, which may assist healthcare professionals in optimizing treatment plans, enhancing overall health outcomes, and improving the quality of life (QoL) of patients with T2DM in India.

## Materials and methods

Study design and patients

This was a retrospective, single-center, observational study that included patients with T2DM aged ≥18, excluding patients with T1DM. This study was conducted at Gupta Ultrasound and Heart Care Centre, New Delhi, India. A consecutive sample of patients (aged ≥18 years) with T2DM who were starting with the use of FreeStyle Libre 2 was included in the study. Only patients who received continuous assistance for DM for at least one year were included in the study. The exclusion criteria were patients with T1DM, gestational DM, and other types of DM. The first application was considered the first use of FreeStyle Libre. The measurements were derived from a sensor that operated continuously for as long as 14 days. The second application meant patients were using FreeStyle Libre for the second time continuously for 14 days following a break of two weeks after the first application. Data from the first application of FreeStyle Libre as well as from a subset of patients who continued using FreeStyle Libre for the second application was collected. The core technology of the FreeStyle Libre system remains consistent between the first and second applications. The first application provides initial insights into glucose trends, allowing healthcare providers to adjust treatment, while the second application helps assess the effectiveness of previous treatment adjustments, identifying any necessary refinements.

The data was retrospectively collected from March 1, 2024, to May 30, 2024, from medical charts. This study was entirely observational, meaning that no interventions were administered during the study. Patients who were previously treated with interventions such as oral anti-diabetic regimens and insulin continued the treatment throughout this study. Oral medications are more convenient but may become less effective over time, particularly as insulin deficiency progresses in T2DM. The patients were given an educational package and training regarding how to use the device [12].

The study was conducted in compliance with the International Conference on Harmonization Good Clinical Practice guidelines and the principles of the Declaration of Helsinki. The Institutional Review Board and Ethics Committee of the Good Society for Ethical Research (approval number: GSER/2023/BMR-AP/079) approved the study protocol. Informed consent from patients was waived because this study utilized deidentified and anonymized data.

Outcomes

Time-in-range (TIR) is the amount of time those with DM spend with their blood glucose levels in a recommended target range and is represented as a percentage (70 and 180 mg/dL). Time-below-range (TBR) is the time spent with blood sugar lower than the recommended range (54 and 70 mg/Dl). Time-above-range (TAR) is the time spent above the recommended range (>140 mg/dL) [13].

The primary outcome of this study was to investigate the correlations between TIR and various T2DM demographic and CGM metrics. CGM values were recorded during the first and second applications of FreeStyle Libre devices for patients using CGM devices for >14 days. The demographic data, along with HbA1c values for each patient, average blood glucose (ABG) levels, TIR, TBR, and TAR, were collected. This study also obtained the details regarding the oral and interventional T2DM medication regimens, duration of T2DM, and comorbidity data.

Sample size

A total of 845 adult patients with T2DM were screened for eligibility. Among them, 650 patients who fulfilled the inclusion and exclusion criteria were included in the final analysis [14].

Statistical analysis

Data were mostly non-normally distributed (as determined by the Shapiro-Wilk test) and are presented as mean (standard deviation). Spearman's rank correlation coefficient was used to assess correlations between %TIR and other variables such as age, gender, body mass index (BMI), HbA1c, ABG, %TBR, and %TAR. Linear regression and multiple regression analyses were performed using %TIR as the objective variable and age, gender, BMI, HbA1c, ABG, %TBR, and %TAR as explanatory variables. Missing CGM data were not replaced. P-values of <0.05 were considered statistically significant, and all tests were two-sided. Statistical analyses were performed using the R software (Version 4.0.3, R Foundation for Statistical Computing, Vienna, Austria (https://www.R-project.org/)). As this was a feasibility study, no prior power calculation was carried out.

## Results

Patient demographics

A total of 649 and 60 patients were included from the first application and second application of FreeStyle Libre, respectively. Male patients constituted 50.69% and 56.67% from the first application and second application, respectively. Most patients from both cohorts (first application, 70.7%; second application, 53.3%) reported an average duration of DM between 10 and 15 years, indicating that a substantial portion of the population has been managing DM for a significant period. Regarding comorbidities, hypertension was the most reported: 93.1% of patients from the first application cohort and 80% of patients from the second application cohort. Other comorbidities in the first application cohort included coronary artery disease (5.5%), chronic kidney disease (2.5%), and diabetic peripheral neuropathy (0.9%). The second application cohort reported neither new comorbidities nor cured/resolved comorbid diseases for any patients. A subgroup analysis was conducted between genders for the first application, which showed a difference in clinical indicators such as weight, HbA1c level, average glucose, %TIR, and %TAR (Table 1). Statistical significance was observed for %TIR between male and female subsets (Appendix A). Similarly, demographic results were compared for the first and second application cohorts where statistical significance was found in %TIR, %TAR, and ABG values. Overall, age and BMI measures did not significantly change over the time between both applications (Table 2).

**Table 1:** Descriptive statistics of all samples and subgroups based on gender for the first application BMI: body mass index; SD: standard deviation; TAR: time-above-range; TBR: time-below-range; TIR: time-in-range Statistical test: Mann-Whitney U test. Normal levels of HBA1c for adults: less than 36 mmol/mol. Normal levels of HBA1c for patients with diabetes: 48 mmol/mol. Normal level of average glucose for adults: less than 100 mg/dL

Variable (mean±SD)	All samples	Female (N=320)	Male (N=329)	P-value
Age (years)	58.82±12.65	60.08±10.3	57.6±14.49	0.060
BMI (kg/m^2^)	24.71±8.29	24.83±11.15	24.6±3.83	0.144
HBA1c (mmol/mol)	8.058±1.82	8.225±1.86	7.895±1.77	0.014
Average glucose (mg/dL)	171.7±51.14	178.4±51.57	165.3±49.96	<0.001
%TIR	42.63±26.55	38.42±25.79	46.72±26.68	<0.001
%TBR	6.339±9.4	6.769±9.49	5.921±9.3	0.096
%TAR	51±29.13	54.74±28.6	47.37±29.22	0.002

**Table 2:** Descriptive statistics for subgroups based on the first and second applications of FreeStyle Libre BMI: body mass index; SD: standard deviation; TAR: time-above-range; TBR: time-below-range; TIR: time-in-range Statistical test: Mann-Whitney U test

Variable (mean±SD)	First application (N=649)	Second application (N=60)	P-value
Age (years)	59.67±12.64	59.67±12.64	-
BMI (kg/m^2^)	24.5±3.22	24.5±3.22	-
HBA1c (mmol/mol)	8.55±2.34	8.57±1.99	1
Average glucose (mg/dL)	173.7±52.96	153.4±30.46	0.006
%TIR	40.82±24.27	64.48±21.78	<0.001
%TBR	5.67±9.70	5.17±8.83	0.766
%TAR	53.52±27.16	30.30±22.16	<0.001

Correlation analysis

For the first application, correlation analysis using Spearman's test revealed a weak positive correlation of the following parameters compared with TIR: TBR (r=0.190; p<0.001). A negative correlation with TIR was observed in patients with lower HbA1c levels (r=−0.547; p<0.001), lower average glucose levels (r=−0.790; p<0.001), and TAR (r=−0.951; p<0.001) (Table 3). Comparison of correlation of variables with TIR from the first and second applications was as follows: a very strong negative correlation was observed for %TAR (p<0.001) and average glucose level (p<0.001). In addition, a moderate negative and statistically significant correlation was identified for HbA1c (p=0.0001) during the first application. A very strong negative correlation of %TAR (p<0.001) and a moderate to strong negative correlation of average glucose level (p<0.001) during the second application indicate their inverse relationship with %TIR (Table 4). Compared with the first sensor application, an increase in correlation values was observed for age, BMI, and HbA1c variables and was not statistically significant, whereas a decrease in TBR value was observed in association with TIR. This indicates that durable TIR achieved with the assistance of CGM improves glycemic control in patients with DM in this study.

**Table 3:** Correlation coefficient of %TIR with other variables in all samples and in subgroups based on gender for the first application *p<0.05; **p<0.01; ***p<0.001 BMI: body mass index; TAR: time-above-range; TBR: time-below-range; TIR: time-in-range

Parameters	All samples	Female (N=320)	Male (N=329)
Age (years)	0.061	0.041	0.098
BMI (kg/m^2^)	0.021	0.061	-0.026
HBA1c (mmol/mol)	-0.547**	-0.561**	-0.527**
Average glucose (mg/dL)	-0.790**	-0.806**	-0.766**
%TBR	0.190**	0.237**	0.163**
%TAR	-0.951**	-0.952**	-0.943**

**Table 4:** Correlation coefficient of %TIR compared between the first and second applications of FreeStyle Libre *p<0.05; **p<0.01; ***p<0.001 BMI: body mass index; TAR: time-above-range; TBR: time-below-range; TIR: time-in-range

Parameters	First application	Second application
Age	-0.217	-0.061
BMI	-0.145	0.04
HBA1c	-0.475***	0.072
Average glucose	-0.759***	-0.687***
%TBR	0.171	0.005
%TAR	-0.936***	-0.889***

Regression analysis

**Figure 1:**
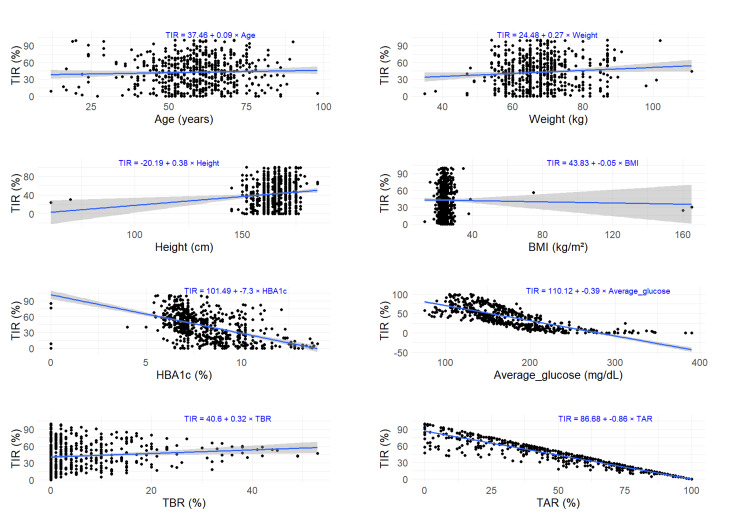
Regression plots of %TIR versus various parameters during the first application: (A) age (years); (B) weight; (C) height; (D) BMI; (E) HBA1c; (F) average glucose; (G) %TBR; and (H) %TAR (the gray zone indicating 95% confidence interval for predictions) BMI: body mass index; TAR: time-above-range; TBR: time-below-range; TIR: time-in-range

**Figure 2:**
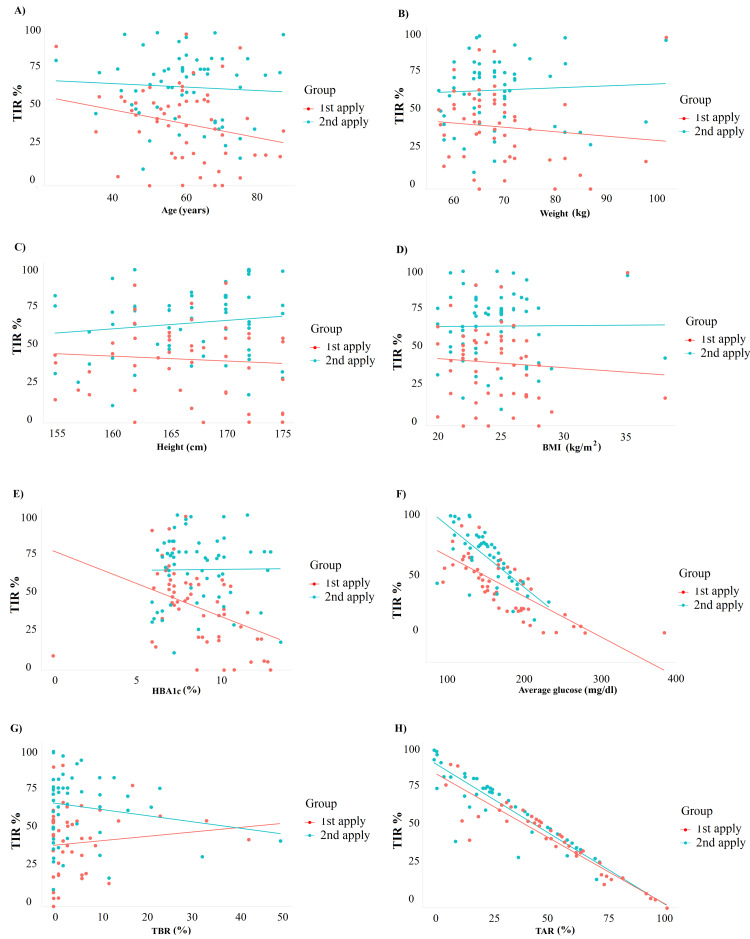
Regression plots of %TIR versus various parameters for comparison between the first and second applications: (A) age (years); (B) weight; (C) height; (D) BMI; (E) HBA1c; (F) average glucose; (G) %TBR; and (H) %TAR BMI: body mass index; TAR: time-above-range; TBR: time-below-range; TIR: time-in-range

On applying single linear regression models, a positive change was observed in TIR for one unit change of each, age (0.088), weight (0.268), height (0.38), and TBR (0.32) for the first application. All parameters showed statistical significance except for age and BMI (Table 5). The regression plots for each of the parameters are illustrated in Figure 1. For the comparative analysis of the first and second applications, statistical significance was observed in HbA1c, average glucose, and TAR for the first application and average glucose and TAR in the second application (Table 6). The regression plots for each of the parameters are illustrated in Figure 2. The results of the linear regression model replicate the correlation results, and a higher and stable %TIR is maintained in most variables except ABG and TAR during the second sensor application, which may reflect the tight glycemic control in these patients.

**Table 5:** Linear regression of %TIR with other variables during the first application of FreeStyle Libre *p<0.05; **p<0.01; ***p<0.001 BMI: body mass index; TAR: time-above-range; TBR: time-below-range; TIR: time-in-range

Parameter	Coefficient	P-value
Age (years)	0.088	0.286
BMI (kg/m^2^)	-0.052	0.679
HBA1c (mmol/mol)	-7.305	<0.001
Average glucose (mg/dL)	-0.393	<0.001
%TBR	0.32	<0.01
%TAR	-0.864	<0.001
Male gender	8.305	<0.001

**Table 6:** Comparison of linear regression of %TIR with other variables between the first and second applications of FreeStyle Libre BMI: body mass index; TAR: time-above-range; TBR: time-below-range; TIR: time-in-range

Variables	First application		Second application	
	Coefficient	P-value	Coefficient	P-value
Age (years)	-0.455	0.068	-0.113	0.62
BMI (kg/m^2^)	-0.541	0.586	0.061	0.945
HBA1c (mmol/mol)	-4.172	<0.01	0.126	0.93
Average glucose (mg/dL)	-0.338	<0.001	-0.515	<0.001
%TBR	0.291	0.377	-0.395	0.221
%TAR	-0.836	<0.001	-0.904	<0.001
Male gender	-2.699	0.673	7.64	0.18

**Figure 3:**
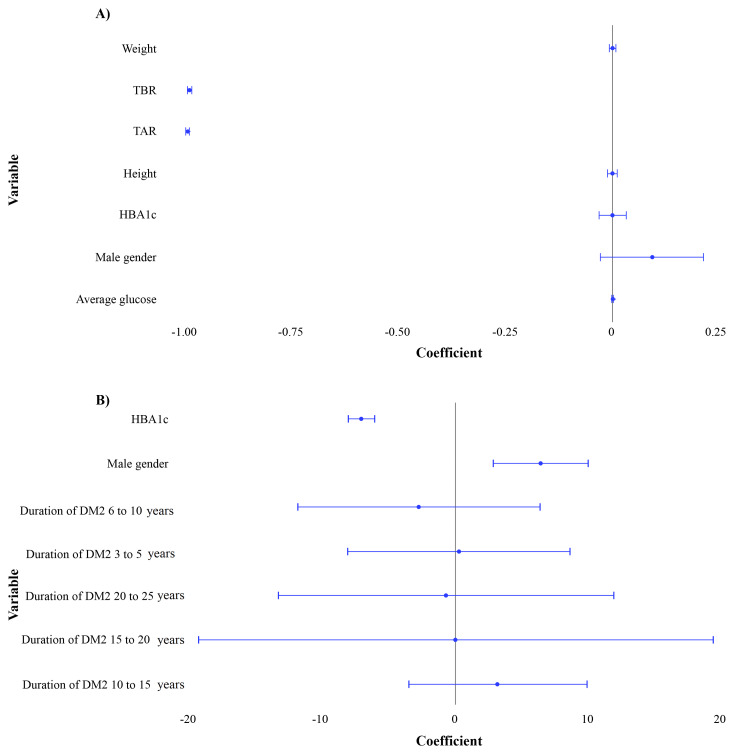
Forest and residual plots for the first application: (A) model 1 (multivariate regression analysis for the first application) and (B) model 2 (multivariate regression analysis for the second application)

Multiple regression models applied to the first application revealed TBR, TAR, and the intercept to be highly significant predictors of TIR in this first model, whereas weight, height, HbA1c, and average glucose did not show statistically significant effects. The second model revealed male patients and HbA1c levels as significant predictors of TIR, while the duration of DM did not show any statistical significance. The forest and residual plots for the first and second models are illustrated in Figure 3. The results are detailed in Table 7. Multiple regression models applied to the comparative analysis of the first and second applications revealed TAR and average glucose as the strongest predictors of TIR. However, multicollinearity was observed between both parameters (first application: r=0.852; p<0.001; second application: r=0.869; p<0.001). Hence, a regression model with TIR as the dependent variable and TAR, HbA1c, and average glucose as the independent variables for each application was applied. For both variables, a statistical significance was observed, providing strong evidence of a significant relationship between TIR and the combination of HbA1c, average glucose, and TAR (Table 8). The visualization of TIR and HbA1c between both applications is illustrated in Appendix B. The strong relationship between CGM-related metrics TIR and TAR, HbA1c, and average glucose indicates that TIR and TAR are reflective of HbA1c values, provided the levels of HbA1c have been stable for the past several months.

**Table 7:** Multiple regression models showing the comparison of %TIR with other variables during the first application of FreeStyle Libre DM: diabetes mellitus; TAR: time-above-range; TBR: time-below-range Model 1 (multivariate regression analysis for the first application). Model 2 (multivariate regression analysis for the second application)

Parameter	Coefficient	P-value
Model 1		
HBA1c (%)	0.001	0.938
Average glucose (mg/dL)	0	0.872
%TBR	-0.995	<0.001
%TAR	-1	<0.001
Male gender	0.093	0.135
Model 2		
Male gender	6.464	<0.001
Duration of DM 10-15 (years)	3.201	0.352
Duration of DM 15-20 (years)	0.027	0.998
Duration of DM 20-25 (years)	-0.693	0.914
Duration of DM 3-5 (years)	0.296	0.945
Duration of DM 6-10 (years)	-2.755	0.554
HBA1c (%)	-7.062	<0.001

**Table 8:** Multiple regression model showing the comparison between the first and second applications of FreeStyle Libre TAR: time-above-range

	Coefficient	P-value
First application		
HBA1c+average glucose (during first application)	0.039	0.298
TAR (during first application)	-0.899	<0.001
Second application		
HBA1c+average glucose (during second application)	0.305	<0.001
TAR (during second application)	-1.277	<0.001

## Discussion

In this study, we have demonstrated the performance and association of FreeStyle Libre with changes in CGM metrics in patients with T2DM from India. Our results showed a consistent negative correlation of TIR with average glucose and TAR, irrespective of the first or second application of FreeStyle Libre. Furthermore, we observed a strong relationship between TIR and the combination of HbA1c and average blood glucose.

The incidence of T2DM is increasing at an alarming rate, especially in low- and middle-income countries. In India, this pertinent issue is restricted to not only low-income rural areas but also the urban middle class, owing to various factors such as a sedentary lifestyle, diet, lack of proper exercise, and so on. Moreover, approximately 70% of patients with T2DM have poor glycemic control (i.e., HbA1c >50 mmol/mol). In recent years, increased awareness of glucose monitoring encouraged the use of CGM systems rather than self-monitoring blood glucose (SMBG), especially in patients with T1DM and T2DM. FreeStyle Libre systems were one of the CGM sensor devices that were introduced into the market, and they were upgraded with greater accuracy evolving to replace the use of conventional monitoring systems. Several observational studies have reported the use of FreeStyle Libre that is associated with a decrease in HbA1c levels, severe hypoglycemic events, and the incidence of diabetic ketoacidosis [8,9].

The value of HbA1c remains the gold standard metric for assessing long-term glycemic levels and represents mean blood glucose levels over the past two to three months [15]. Improved glucose monitoring could significantly prevent the development of diabetic-associated complications such as diabetic neuropathy [16,17], as fluctuations in glucose levels could lead to more serious damage to nerves than in controlled hyperglycemia [17].

TIR represents the blood glucose levels for the past 72 hours and is one of the key indicators in the assessment of blood glucose, with a better linear correlation with HbA1c [18]. Similar to HbA1c, TIR could also be an indicator for detecting and predicting the microvascular diabetic complications. Studies reported that at least a 5% increase in TIR could significantly benefit clinically in patients with T2DM [19]. Studies based on CGM in insulin-treated patients with T2DM either failed to report improvement in HbA1c or showed modest glycemic benefits. Our study showed a significant reduction in HbA1c levels without affecting hypoglycemic exposure [20,21]. A meta-analysis of 75 real-world evidence showed that the FreeStyle Libre system reduces HbA1c for adults with both T1DM and T2DM significantly. These reductions are more pronounced in individuals with higher baseline HbA1c levels and are sustained for 24 months in T1DM and 12 months in T2DM [22].

The ALERTT1 trial compared real-time CGM (rtCGM) with intermittently scanned CGM (isCGM) in adults with T1DM. The results showed significantly improved TIR (59.6% vs. 51.9%) and reduced HbA1c (7.1% vs. 7.4%), suggesting that switching to rtCGM can improve health and QoL for people with DM. Additionally, hypoglycemia, defined as glucose levels below 3.0 mmol/L, was also reduced for rtCGM users at 0.47%, compared to 0.84% for those on isCGM. Furthermore, participants using rtCGM reported less worry about hypoglycemia and experienced fewer instances of severe hypoglycemia [23]. Failure of beneficial associations between TIR and HbA1c in previous studies might be due to high baseline HbA1c levels of >8.0 or involvement of patients who had multiple failed non-insulin therapies [20,21].

The study showed a strong proportional relation between TIR and HbA1c levels, average glucose levels, and TAR. Moreover, a significant increase in %TIR was found in both the first (40.8%) and the second (64.4%) applications, whereas a significant decrease in %TAR was observed. TBR reductions at the initial stages play a prominent role in reducing hypoglycemia and related complications [19]. A single-center, retrospective GLITTER study involving 105 adult patients with T2DM showed an improved TIR (i.e., 42-80%) with a significant decrease in TAR (52% to 18%) and TBR (5.7% to 1.5%) values [24]. Our study also showed a significant increase in TIR (i.e., 40.8-64.4%) but only a slight decrease in TBR (i.e., 5.76% to 5.17%).

Dietary differences of the patients and type of diabetic treatment/medications between the studies might have influenced the modest decrease in TBR. However, the increase in TIR in our study is highly welcoming, and the use of medications such as glucagon-like peptide 1 (GLP-1) receptor agonists and sodium-glucose co-transporter 2 (SGLT-2) inhibitors was reported to improve TIR [25,26]. Although most patients in our study were under treatment with GLP-1 receptor agonists and SGLT-2 inhibitors, very few patients were also under the insulin treatment.

BMI is one of the indicators for the risk of obesity-induced T2DM [27,28]. An increase in body fat improves the incidence of T2DM and is negatively correlated with TIR. The simple linear regression analysis showed that except age and BMI, the remaining factors are significantly influenced by %TIR (p<0.001), especially in the first application. The linear regression of TIR versus weight indicated that negative changes in TIR are associated with an increase in weight. Furthermore, obesity-associated conditions possess certain adipokines that improve the T2DM condition [29,30]. In our study, the multiple linear regression analysis showed significant associations of TIR with TBR and TAR in the first application and male patients and HbA1c levels in the second application. Moreover, TAR and average glucose levels were considered the strongest predictors of TIR. Several studies have shown that TIR may serve as a potential predictor of future diabetic complications in both T1DM and T2DM [31,32]. A study by Beck et al. observed that a 10% increase in TIR70-180 (approximately 2.4 hours per day) was associated with an average decrease in HbA1c of 0.6%, highlighting the significant role of TIR in influencing HbA1c levels [33].

In a study by Alazmi et al., HbA1c showed a statistically significant correlation with both TIR and glycemic management indicator, with correlation coefficients (r) of 0.78 (p<0.001) and 0.82 (p<0.001), respectively. Additionally, a linear regression model between TIR and HbA1c was developed, which was statistically significant (p<0.001) [34].

The strength of this study is that it combines real-time glycemic data from CGM devices (FreeStyle Libre) with HbA1c, offering a more holistic and actionable approach to managing T2DM. This improves clinical outcomes and enhances the patient's QoL by enabling more personalized and effective treatment strategies. However, the study has certain limitations such as single-center analysis and the absence of information on anemia or genetic variations of patients, which could influence the diabetic conditions. Due to the retrospective nature of the study, selection bias is also a limitation. To mitigate this, we used multivariable regression to adjust for potential confounders.

## Conclusions

Overall, the use of the CGM device FreeStyle Libre for data, such as TIR, TBR, and TAR, along with HbA1c could be proven effective for glycemic control in patients with DM. In addition, CGM metrics might assist healthcare professionals in optimizing treatment plans and improving overall health outcomes, thereby reducing the number of DM-related complications, which in turn could assist in improving the clinical and QoL outcomes in patients with T2DM.

## Figures and Tables

**Figure 4:**
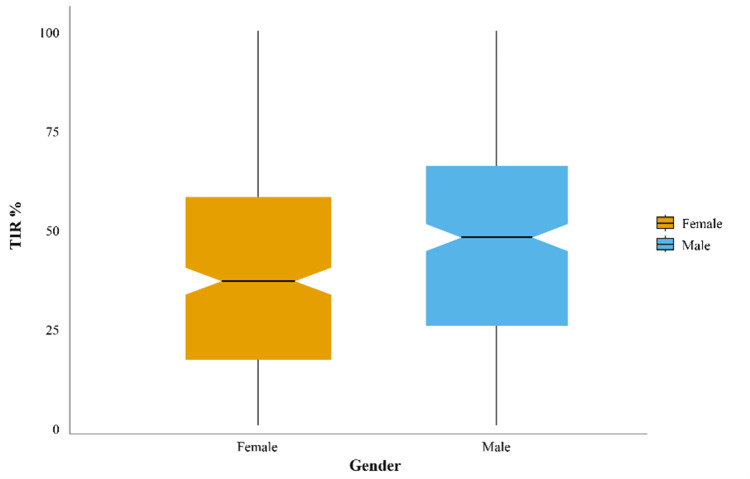
Boxplot describing the distribution of the %TIR variable in males and females TIR: time-in-range

**Figure 5:**
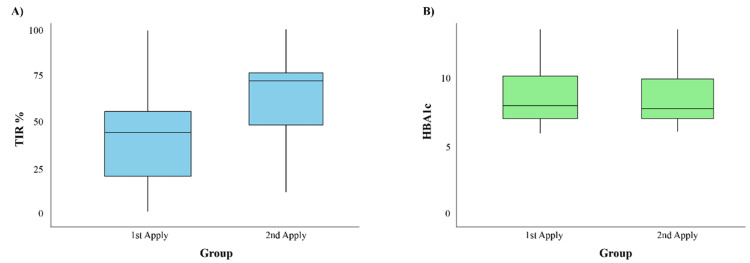
Visualization of %TIR and HbA1c between both applications: (A) boxplot of %TIR and (B) boxplot of HBA1c TIR: time-in-range

## Appendices

Appendix A

Appendix B
